# Supplementary material for: Mucosal delivery of a multistage subunit vaccine promotes development of lung-resident memory T cells and affords interleukin-17-dependent protection against pulmonary tuberculosis
Source: NPJ Vaccines. 2020 Nov 12;5:105. doi: 10.1038/s41541-020-00255-7 (PMC7665186; doi:10.1038/s41541-020-00255-7)
Supplement: Supplementary file 1 — Supplementary Information [file 41541_2020_255_MOESM1_ESM.pdf]

# Supplementary Information

# Mucosal delivery of a multistage subunit vaccine promotes development of lung-resident memory T cells and affords interleukin-17-dependant protection against pulmonary tuberculosis

Claudio Counoupas, Kia Ferrell, Anneliese Ashhurst, Nayan D. Bhattacharyya, Gayathri Nagalingam, Erica Stewart, Carl G. Feng, Nikolai Petrovsky, Warwick J. Britton, James A. Triccas

## Table of Contents

**Supplementary Figure 1. Gating strategy for $CD4^+$ cells expression of cytokines and transcription factors.**

*Page 1*

**Supplementary Figure 2. Comparative analysis of multifunctional $CD4^+$ T cell subsets before and after *M. tuberculosis* infection of mice vaccinated with CysVac2/Advax$^{CpG}$ either via pulmonary or parenteral route.**

*Page 2*

**Supplementary Figure 3. Comparative analysis of lung multifunctional $CD4^+$ T cell subsets after *M. tuberculosis* infection of mice vaccinated via pulmonary route with either CysVac2/Advax or CysVac2/Advax$^{CpG}$.**

*Page 3*

**Supplementary Figure 4. tSNE expression of markers in the lung of mice after *M. tuberculosis* infection and anti-IL-17 mAb treatment.** *Page 4*

**Supplementary Figure 5. Effects of blocking IL-17 during *M. tuberculosis* infection on $CD4^+$ T cells proliferation in the lung.** *Page 5*

**Supplementary Table 1. List of antibodies used** *Page 6*

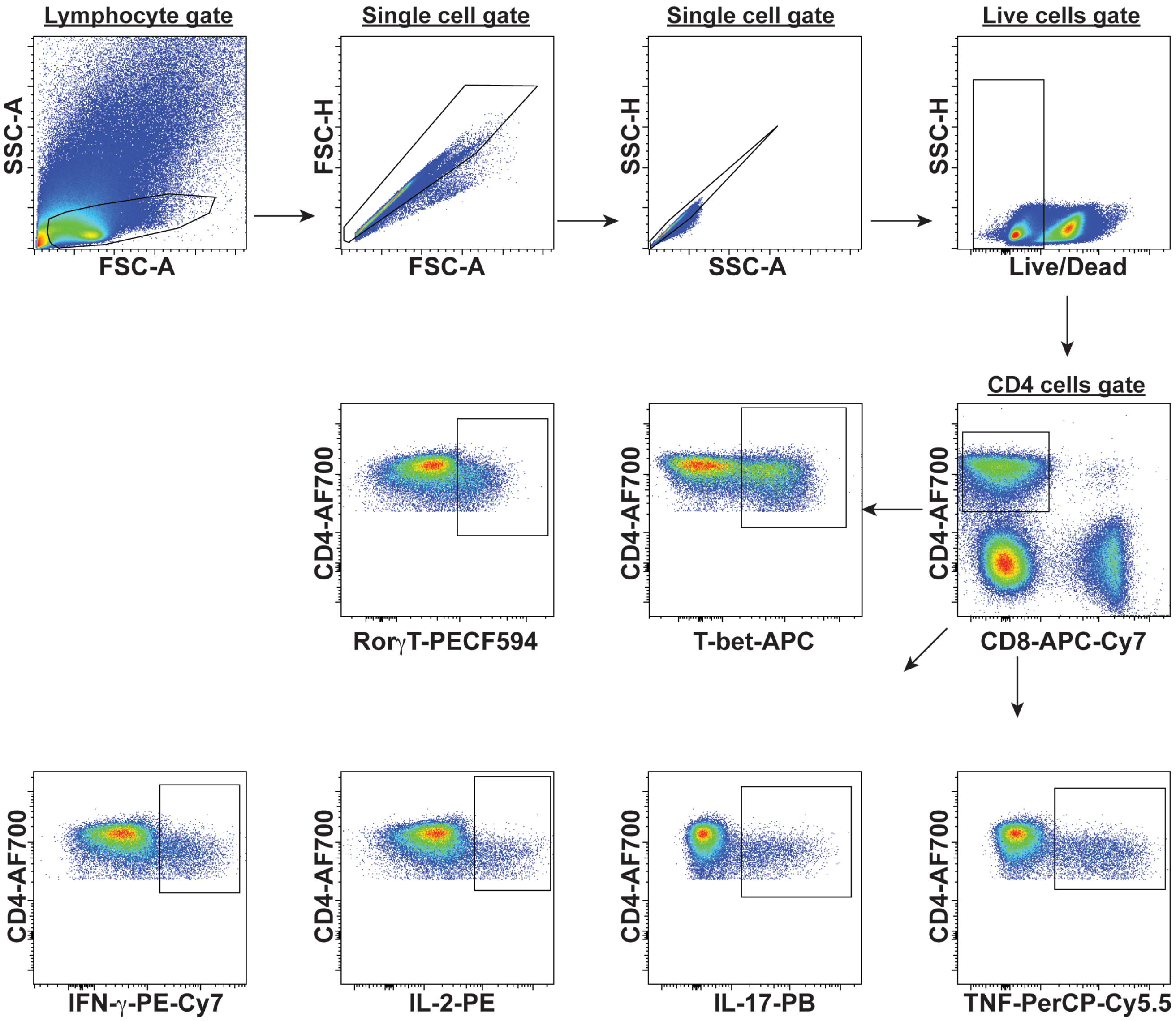


**Supplementary Figure 1. Gating strategy for CD4$^+$ cells expression of cytokines and trascription factors.** Lymphocytes populations were gated by SSC-A and FSC-A plots. Single events were selected using FSC-H/FSC-A and SSC-A/SSC-H. Dead cells were excluded and viable CD4$^+$ cells were gated for further analysis of expression of different cytokines (IFN-γ, IL-2, IL-17, TNF) and transcrption factors (T-bet and RorγT,).

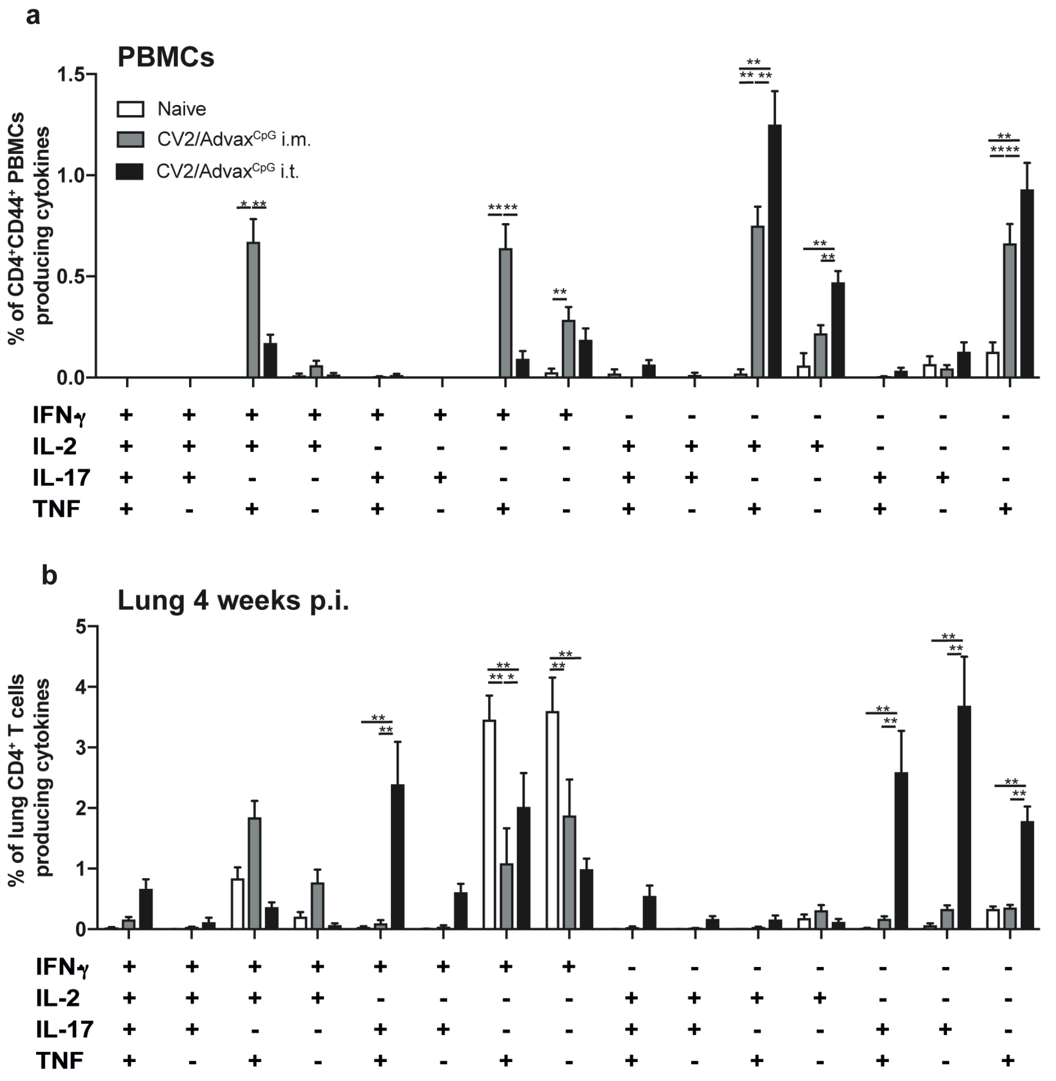


**Supplementary Figure 2. Comparative analysis of multifunctional CD4$^+$ T cell subsets before and after *M. tuberculosis* infection of mice vaccinated with CysVac2/Advax$^{CpG}$ either via pulmonary or parenteral route.**

C57BL/6 mice (n=5-6) were vaccinated by either the i.m. or i.t. route with CysVac2 (CV2)/Advax-$^{CpG}$ (3 times, 2 weeks apart). One week after last vaccination mice were bled for vaccine immunogenicity assessment. Six weeks after last immunization mice were challenged with H37Rv by aerosol (~100 CFU) and four weeks later culled to enumerate bacterial burden and T cell phenotype in the lung. PBMCs (one week after last vaccination, panel a) and lung cells 4-weeks p.i. (panel b) were restimulated with CysVac2 fusion protein, and analysed for intracellular expression of IFN-γ, IL-2, IL-17, and TNF by flow cytometry. Boolean gating analysis was performed to identify subsets of CD4$^+$ T cells expressing different combinations of these cytokines. Data are represented as the percentage of cytokine-producing CD4$^+$ T cells ± SEM and is representative of 2 independent experiments. Significance difference between the groups was determined by ANOVA (*p<0.05; **p<0.01).

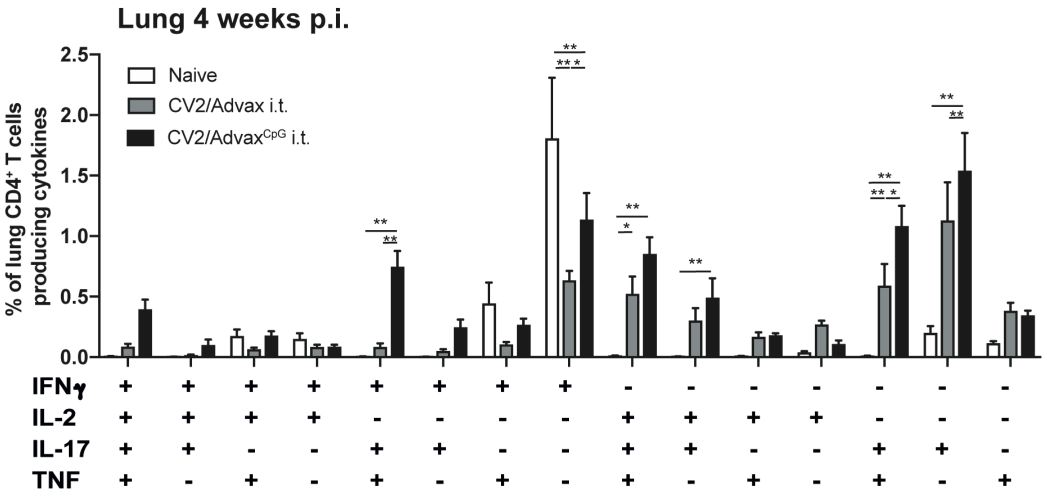


**Supplementary Figure 3. Comparative analysis of lung multifunctional CD4$^{+}$ T cell subsets after *M. tuberculosis* infection of mice vaccinated via pulmonary route with either CysVac2/Advax or CysVac2/Advax$^{CpG}$.**

C57BL/6 mice (n=5-6) were vaccinated by i.t. route with either CysVac2 (CV2)/Advax or CysVac2 (CV2)/Advax$^{CpG}$ (3 times, 2 weeks apart). Six weeks after last immunization mice were challenged with H37Rv by aerosol (~100 CFU), and four weeks later culled to enumerate bacterial burden and T cell phenotype in the lung. Lung cells 4 weeks p.i. were restimulated with CysVac2 fusion protein, and analysed for intracellular expression of IFN-γ, IL-2, IL-17, and TNF by flow cytometry. Boolean gating analysis was performed to identify subsets of CD4$^{+}$ T cells expressing different combinations of these cytokines. Data are represented as the percentage of cytokine-producing CD4$^{+}$ T cells ± SEM and is representative of 2 independent experiments. Significance difference between the groups was determined by ANOVA (*$p < 0.05$; **$p < 0.01$).

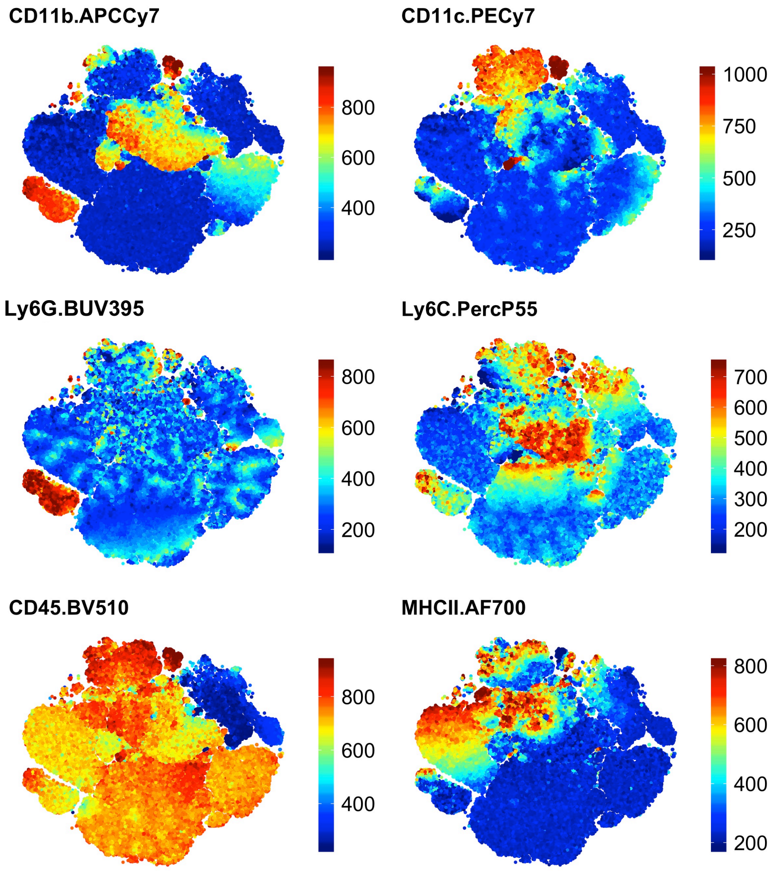


**Supplementary Figure 4. tSNE expression of markers in the lung of mice after *M. tuberculosis* infection and anti-IL-17 mAb treatment.** Example of tSNE dimension 1 and 2 plots of the lung compartment show relative expression intensity of each indicated phenotypic marker. tSNE heat maps show fluorescent intensity of each marker for each event. Scales on the heat maps are individually generated for each surface marker from low to high expression. (Ashhurst, T. M. 2017, tSNEplots v1.3. GitHub repository).

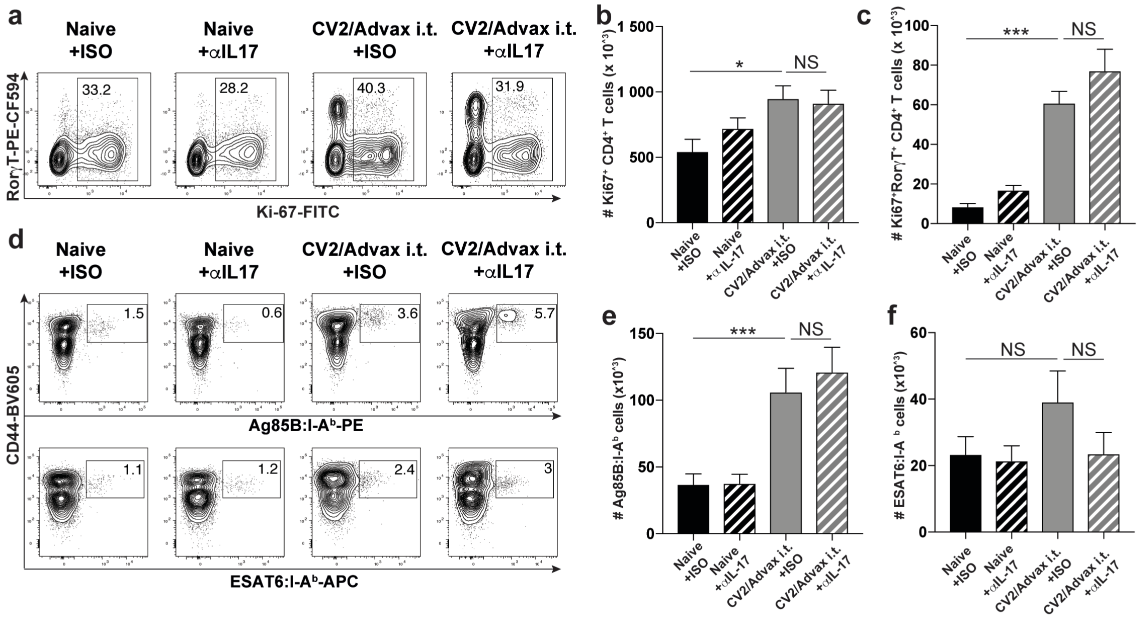


**Supplementary Figure 5. Effects of blocking IL-17 during *M. tuberculosis* infection on CD4+ T cells proliferation in the lung.**

C57BL/6 mice (n=5-6) were vaccinated i.t. with CysVac2/Advax and treated i.p. with anti-IL-17 mAb, as described in Figure 5. Representative dot plot of the expression of Ki67 and RORγT on CD4+ T cells from the lung (a). Bar graphs showing numbers of total (b) and RorgT+ (c) proliferating CD4+ T cells enumerated in the lung. Representative dot plots show CD44 and either Ag85B:I-Ab or ESAT6:I-Ab staining on CD4+ T cells in the lung (d), with total number ± SEM of Ag85B:I-Ab+ (e) and ESAT6:I-Ab+ (f) CD4+ T cells in the lung. Data are pooled of 2 independent experiments and represent average number of cells ± SEM. Significance of differences between the groups was determined by ANOVA (*p<0.05; ***p<0.001; NS=not significant).

**Supplementary Table 1. List of antibodies used**

| Marker | Fluorophore | Clone | Dilution | Company | Cat. # |
|---|---|---|---|---|---|
| CD103 | BV786 | M290 | 1:100 | BD | 564322 |
| CD11a | BV510 | M17/4 | 1:100 | BD | 563669 |
| CD11b | APC-Cy7 | M1/70 | 1:100 | BD | 557657 |
| CD11c | AF700 | N418 | 1:200 | Biolegend | 117320 |
| CD4 | AF700 | RM414 | 1:200 | BD | 557956 |
| CD44 | BV605 | IM7 | 1:300 | BD | 563058 |
| CD45 | Biotin | 104 | 1:200 | BD | 553771 |
| CD45 | BV510 | 104 | 1:200 | BD | 740131 |
| CD62L | eFluor450 | MEL-14 | 1:200 | eBioscience | 48-0621-82 |
| CD64 | PECy7 | X54-5/7.1 | 1:200 | BioLegend | 139313 |
| CD69 | FITC | H1.243 | 1:200 | BD | 557392 |
| CD8 | APCy7 | 53-6.7 | 1:200 | BD | 557654 |
| CD80 | BV450 | 16-1081 | 1:200 | BD | 560523 |
| CD86 | FITC | GL-1 | 1:200 | Biolegend | 105006 |
| Fc Block | purified | 2462 | 1:300 | BD | 553141 |
| IFN-γ | PECy7 | XMG1-2 | 1:200 | BD | 557649 |
| IL-17 | PB | TC11-18H10.1 | 1:200 | BioLegend | 506918 |
| IL-2 | PE | JES6-5H4 | 1:200 | BD | 554428 |
| KLRG-1 | PE-Cy7 | 2F1 | 1:200 | BD | 561621 |
| Ki-67 | FITC | SP6 | 1:200 | eBioscience | 11-5698-82 |
| Ly6C | PerCP-Cy5.5 | HK1.4 | 1:200 | eBioscience | 45-5932-82 |
| Ly6G | BUV395 | 1A8 | 1:200 | BD | 565964 |
| MHCII | BV421 | M5/114.15.2 | 1:300 | Biolegend | 107632 |
| MHCII | AF700 | M5/114.15.2 | 1:200 | Biolegend | 107622 |
| NK1.1 | BV785 | PK136 | 1:100 | Biolegend | 108749 |
| PD-1 | BV711 | 29F.1A12 | 1:200 | Biolegend | 135231 |
| RorγT | PE-CF594 | Q31378 | 1:200 | BD | 562684 |
| SiglecF | PE | E50-2440 | 1:200 | BD | 552126 |
| Streptavidin | APC-Cy7 |  | 1:200 | BD | 554063 |
| T-bet | APC | 4B10 | 1:200 | BioLegend | 644814 |
| TNF | PerCP-Cy5.5 | MP6-XT22 | 1:200 | BD | 560659 |
