# Supplementary material for: Mucosal delivery of a multistage subunit vaccine promotes development of lung-resident memory T cells and affords interleukin-17-dependent protection against pulmonary tuberculosis
Source: NPJ Vaccines. 2020 Nov 12;5:105. doi: 10.1038/s41541-020-00255-7 (PMC7665186; doi:10.1038/s41541-020-00255-7)
Supplement: Supplementary file 2 — Reporting Summary Checklist [file 41541_2020_255_MOESM2_ESM.pdf]

nature research

Corresponding author(s): Claudio Counoupas and Jamie Triccas

Last updated by author(s): 13/08/2020

Reporting Summary

Nature Research wishes to improve the reproducibility of the work that we publish. This form provides structure for consistency and transparency in reporting. For further information on Nature Research policies, see our Editorial Policies and the Editorial Policy Checklist.

Statistics

For all statistical analyses, confirm that the following items are present in the figure legend, table legend, main text, or Methods section.

n/a	Confirmed	
☐	☒	The exact sample size (*n*) for each experimental group/condition, given as a discrete number and unit of measurement
☐	☒	A statement on whether measurements were taken from distinct samples or whether the same sample was measured repeatedly
☐	☒	The statistical test(s) used AND whether they are one- or two-sided<br>*Only common tests should be described solely by name; describe more complex techniques in the Methods section.*
☒	☐	A description of all covariates tested
☐	☒	A description of any assumptions or corrections, such as tests of normality and adjustment for multiple comparisons
☐	☒	A full description of the statistical parameters including central tendency (e.g. means) or other basic estimates (e.g. regression coefficient) AND variation (e.g. standard deviation) or associated estimates of uncertainty (e.g. confidence intervals)
☒	☐	For null hypothesis testing, the test statistic (e.g. *F*, *t*, *r*) with confidence intervals, effect sizes, degrees of freedom and *P* value noted<br>*Give P values as exact values whenever suitable.*
☒	☐	For Bayesian analysis, information on the choice of priors and Markov chain Monte Carlo settings
☒	☐	For hierarchical and complex designs, identification of the appropriate level for tests and full reporting of outcomes
☒	☐	Estimates of effect sizes (e.g. Cohen's *d*, Pearson's *r*), indicating how they were calculated

Our web collection on statistics for biologists contains articles on many of the points above.

Software and code

Policy information about availability of computer code

Data collection	FACS Diva v.8<br>Tecan iControl software
Data analysis	GraphPad Prism v.7<br>FlowJo v.10<br>FiJi Imaging Software

For manuscripts utilizing custom algorithms or software that are central to the research but not yet described in published literature, software must be made available to editors and reviewers. We strongly encourage code deposition in a community repository (e.g. GitHub). See the Nature Research guidelines for submitting code & software for further information.

Data

Policy information about availability of data

All manuscripts must include a data availability statement. This statement should provide the following information, where applicable:

- Accession codes, unique identifiers, or web links for publicly available datasets
- A list of figures that have associated raw data
- A description of any restrictions on data availability

All relevant data generated or analysed during this study are included in this published article (and its supplementary information files), raw files can be requested from corresponding authors.

Field-specific reporting

Please select the one below that is the best fit for your research. If you are not sure, read the appropriate sections before making your selection.

☒ Life sciences ☐ Behavioural & social sciences ☐ Ecological, evolutionary & environmental sciences

For a reference copy of the document with all sections, see nature.com/documents/nr-reporting-summary-flat.pdf

Life sciences study design

All studies must disclose on these points even when the disclosure is negative.

Sample size	For all experiments, we have determined the number of animals required based on the ability to detect a 40% difference between groups, a significance of p<0.05 and 80% power to reject the null hypothesis given the alternative hypothesis is true.
Data exclusions	No Data was excluded
Replication	All experiments were performed at least twice.
Randomization	Allocation of mice to experimental groups was randomized upon their arrival to the animal facility.
Blinding	Investigators were not blinded to animal groups due to the nature of vaccine delivery. Groups of mice were allocated to receive particular methods of vaccine delivery and vaccine doses which remained consistent throughout the experiment.

Reporting for specific materials, systems and methods

We require information from authors about some types of materials, experimental systems and methods used in many studies. Here, indicate whether each material, system or method listed is relevant to your study. If you are not sure if a list item applies to your research, read the appropriate section before selecting a response.

Materials & experimental systems

n/a	Involved in the study	
☐	☒	Antibodies
☒	☐	Eukaryotic cell lines
☒	☐	Palaeontology and archaeology
☐	☒	Animals and other organisms
☒	☐	Human research participants
☒	☐	Clinical data
☒	☐	Dual use research of concern

Methods

n/a	Involved in the study	
☒	☐	ChIP-seq
☐	☒	Flow cytometry
☒	☐	MRI-based neuroimaging

Antibodies

Antibodies used

Marker Fluorophore Clone Company
CD103 BV786 M290 BD
CD11a BV510 M17/4 BD
CD11b APC-Cy7 M1/70 BD
CD11c AF700 N418 Biolegend
CD4 AF700 RM414 BD
CD44 BV605 IM7 BD
CD45 Biotin 104 BD
CD45 BV510 104 BD
CD62L eFluor450 MEL-14 eBioscience
CD64 PECy7 X54-5/7.1 BioLegend
CD69 FITC H1.243 BD
CD8 APCy7 53-6.7 BD
CD80 BV450 16-1081 BD
CD86 FITC GL-1 Biolegend
Fc Block purified 2462 BD
IFN-[illegible] PECy7 XMG1-2 BD
IL-17 PB TC11-18H10.1 BioLegend
IL-2 PE MQ1-17H12 BD
KLRG-1 PE-Cy7 2F1 BD
Ki-67 FITC SP6 eBioscience
Ly6C PerCP-Cy5.5 HK1.4 eBioscience
Ly6G BUV395 1A8 BD

MHCII V450 M5/114.15.2 Biolegend
MHCII AF700 M5/114.15.2 Biolegend
NK1.1 BV785 PK136 Biolegend
PD-1 BV711 29F.1A12 BD
Ror?T PE-CF594 Q31378 BD
SiglecF PE E50-2440 BD
Streptavidin APC-Cy7 BD
T-bet APC 4B10 BioLegend
TNF PerCP-Cy5.5 MP6-XT22 BD

Validation	Antibodies were validated before use based on the conditions suggested by the manufacturer

Animals and other organisms

Policy information about studies involving animals; ARRIVE guidelines recommended for reporting animal research

Laboratory animals	Female C57BL/6 (6-8 weeks of age)
Wild animals	Study did not involve wild animals
Field-collected samples	Study did not involve samples collected on the field
Ethics oversight	Sydney Local Health District (SLHD) Animal Ethics and Welfare Committee approved the ethic protocol of the study.

Note that full information on the approval of the study protocol must also be provided in the manuscript.

Flow Cytometry

Plots

Confirm that:

☒ The axis labels state the marker and fluorochrome used (e.g. CD4-FITC).

☒ The axis scales are clearly visible. Include numbers along axes only for bottom left plot of group (a 'group' is an analysis of identical markers).

☒ All plots are contour plots with outliers or pseudocolor plots.

☒ A numerical value for number of cells or percentage (with statistics) is provided.

Methodology

Sample preparation	PBMCs were isolated from whole blood as previously described17. Single cell suspensions were prepared from the lung as previously described17. PE-conjugated Ag85B240-254:I-Ab tetramer and APC-conjugated ESAT61-20:I-Ab tetramer were provided by the NIH Tetramer Core Facility. For staining, cells were incubated with tetramers at 37 oC for 1 hour. Cells were stained using the marker-specific fluorochrome-labeled mAbs indicated in S1 Table. Gating strategy for identification of specific cell populations is shown in Fig S1. To assess antigen-specific cytokine induction by T cells, PBMCs or single-cell suspensions from the lung were stimulated for 4 hours with CysVac2 (5 µg/mL) and then supplemented with brefeldin A (10 µg/mL) for further 10-12 hours. Cells were surface stained with Fixable Blue Dead Cell Stain (Life Technologies) and the marker-specific fluorochrome-labeled antibodies indicated in Supplementary Table. Cells were then fixed and permeabilized using the BD Cytofix/CytopermTM kit according to the manufacturer’s protocol
Instrument	LSR Fortessa
Software	FACS DIVA
Cell population abundance	At least one million events were acquired to account for rare populations.
Gating strategy	Data available on Supplementary Material

☒ Tick this box to confirm that a figure exemplifying the gating strategy is provided in the Supplementary Information.
